# Kisspeptin-10 (KP-10) stimulates osteoblast differentiation through GPR54-mediated regulation of BMP2 expression and activation

**DOI:** 10.1038/s41598-018-20571-2

**Published:** 2018-02-01

**Authors:** Hyo-Eun Son, Kyeong-Min Kim, Eun-Jung Kim, Won-Gu Jang

**Affiliations:** 1 Department of Biotechnology, School of Engineering, Daegu University, Gyeongbuk, 38453 Republic of Korea; 2 Research Institute of Anti-Aging, Daegu University, Gyeongbuk, 38453 Republic of Korea; 3 Department of Immunology, Kyungpook National University School of Medicine, Daegu, 41944 Republic of Korea

## Abstract

Kisspeptin-10 (KP-10) acts as a tumor metastasis suppressor via its receptor, G-protein-coupled receptor 54 (GPR54). The KP-10-GPR54 system plays an important role in embryonic kidney development. However, its function in osteoblast differentiation is unknown. Osteoblast differentiation is controlled by a range of hormones and cytokines, such as bone morphogenetic protein (BMPs), and multiple transcription factors, such as Runt-related transcription factor 2 (Runx2), alkaline phosphatase (ALP), and Distal-less homeobox 5 (Dlx5). In the present study, KP-10-treatment significantly increased the expression of osteogenic genes, including mRNA and protein levels of BMP2, in C3H10T1/2 cells. Moreover, KP-10 induced BMP2-luc activity and increased phosphorylation of Smad1/5/9. In addition, NFATc4 specifically mediated KP-10-induced BMP2 gene expression. However, KP-10 treatment did not induce expression of the BMP2 and Runx2 genes in GPR54^−/−^ cells. To examine whether KP-10 induced secretion of BMP2 to the culture medium, we used the conditioned-medium (C.M) of KP-10 treated medium on C3H10T1/2 cells. Dlx5 and Runx2 expressions were higher in GPR54^−/−^ cells treated with C.M than in those treated with KP-10. These results demonstrate that BMP2 protein has an autocrine effect upon KP-10 treatment. Taken together, these findings suggest that KP-10/GPR54 signaling induces osteoblast differentiation via NFATc4-mediated BMP2 expression.

## Introduction

The Kiss1 gene encodes a premature 145-amino acid protein that is proteolytically cleaved into polypeptides known as kisspeptins (KPs), including KP-54, KP-14, KP-13, and KP-10^1^. KP-10 protein binds to G-protein-coupled receptor 54 (GPR54)^1–3^. Activation of KP-10 suppresses pulmonary human melanoma^1,3,4^, and KP-10 is a metastasis suppressor in breast cancer cells^5–8^. KP-10/GPR54 regulates pubertal development and hypogonadotropic hypogonadism. Loss of KP-10/GPR54 is associated with the absence of sexual maturation and low levels of gonadotropic hormones in humans and mice^9–12^. Moreover, GPR54 regulates the expression of bone morphogenetic protein (BMP) 7 through nuclear factor of activated T-cells (NFAT) c2 and Sp1, and plays an important role in embryonic kidney branching morphogenesis and glomerular development^13^. Interaction of KP-10, estrogen and BMP4 regulates gonadotropin-releasing hormone (GnRH) production in GT1-7 cells^14^.

The NFAT family comprises five subtypes NFATc1, NFATc2, NFATc3, NFATc4, and NAFTc5^15^. NFATs can regulate cytokine gene expression in immune cells^16^ and have essential roles in the regulation of immune system^17^. Calcineurin-NFAT signaling is essential for many stages of vertebrate development^18^. A previous study indicates that NFAT signaling regulates both osteoblasts and osteoclasts^19,20^. RANKL-RANK signaling regulates NFATc1 expression during osteoclast differentiation^21^. In addition, NFATc1 is reported to have crucial roles in the proliferation of osteoblasts. NFATs also function in osteoblasts to regulate factors that are crucial for the recruitment of osteoclast precursors to bone^19,22^.

Osteoblast differentiation is highly regulated by a range of hormones, cytokines and multiple transcription factors^23,24^. Osteoblasts express various phenotypic markers, such as BMPs, Distal-less homeobox 5 (Dlx5), Runt-related transcription factor 2 (Runx2), alkaline phosphatase (ALP), and osteocalcin (OC)^23–25^. Among BMPs, BMP2 is the most effective inducer of osteoblast differentiation. In addition, BMP2 regulates early stage of osteoblast differentiation such as Id1, Dlx5, and Runx2 via activating Smad 1/5/9^26–28^. These factors also stimulate expression of marker genes of the next stage of osteogenesis, including ALP and OC^29–31^.

The role of KP-10 in osteoblast differentiation has not yet been elucidated. In this study, we demonstrate that KP-10 regulates BMP2 expression and activation through the GPR54/NFATc4 signaling cascade, and subsequently increases expression of early stage osteogenic marker genes such as Dlx5 and Runx2 via BMP2-induced Smad1/5/9 phosphorylation.

## Results

### KP-10 induces expression of osteogenic marker genes

**Figure 1:**
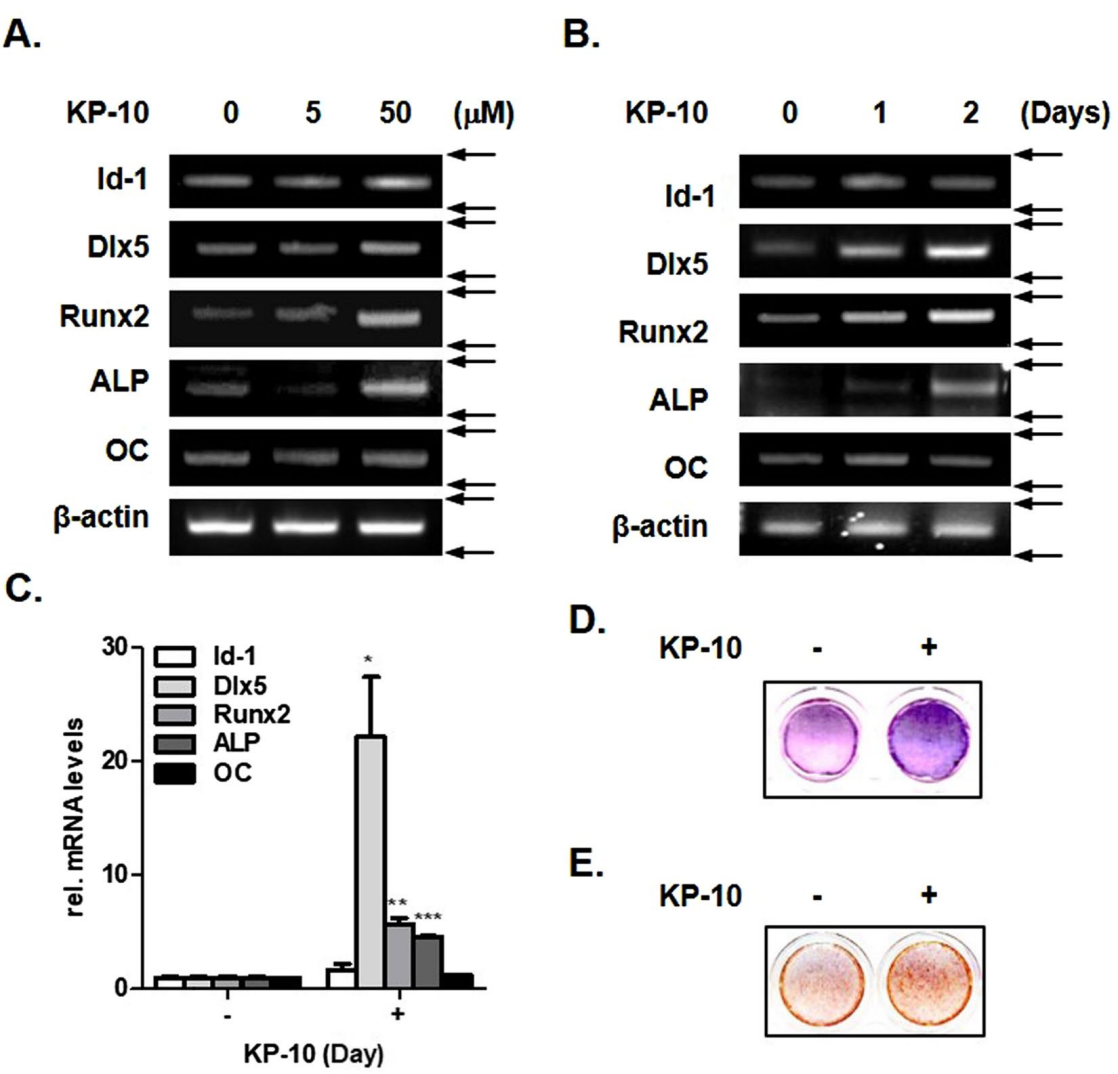
KP-10 induces osteogenic gene expressions in C3H10T1/2 cells. (**A** and **B**) RT-PCR were performed using total RNA isolated from C3H10T1/2 cells treated with 5 or 50 μM KP-10 for 2 days (**A**), 50 μM KP-10 for 1 or 2 days (**B**). (**C**) Real-time PCR were performed using total RNA isolated cells treated with 50 μM of KP-10 for 2 days. *p < 0.05; **p < 0.01 and ***p < 0.005 compared with the untreated control. Data represent the mean ± SEM of three individual experiments. (**D**) Effects of KP-10 on ALP. C3H10T1/2 cells were treated with 50 μM KP-10 in the presence of ascorbic acid and β-glycerophosphate (AA + β-GP) for 4days. (**E**) ARS staining was detected after treating cell with 50 μM KP-10 for 20 days.

To confirm the effect of KP-10 on osteoblast differentiation, we treated C3H10T1/2 cells with 5 and 50 μM KP-10 for 2 days. KP-10 significantly induced expression of osteogenic marker genes in a dose-dependent manner (Fig. 1A). In addition, 50 μM of KP-10 significantly increased expression of osteogenic marker genes in a time-dependent manner (Fig. 1B). Real-time PCR analysis showed that KP-10 also significantly increased expression of osteogenic marker genes (Fig. 1C). Accumulated circumstantial evidence indicates that ALP plays an important role in the formation and calcification of hard tissues^32^. KP-10 significantly increased the staining intensity of ALP (Fig. 1D). In addition, KP-10 induced the mineralization in C3H10T1/2 cells by Alizarin Red S staining (Fig. 1E). Overall, these results suggest that KP-10 increased expression of osteoblast marker genes such as Dlx5, Runx2 and ALP.

### KP-10 induces BMP2 expression and Smad phosphorylation in C3H10T1/2 cells

**Figure 2:**
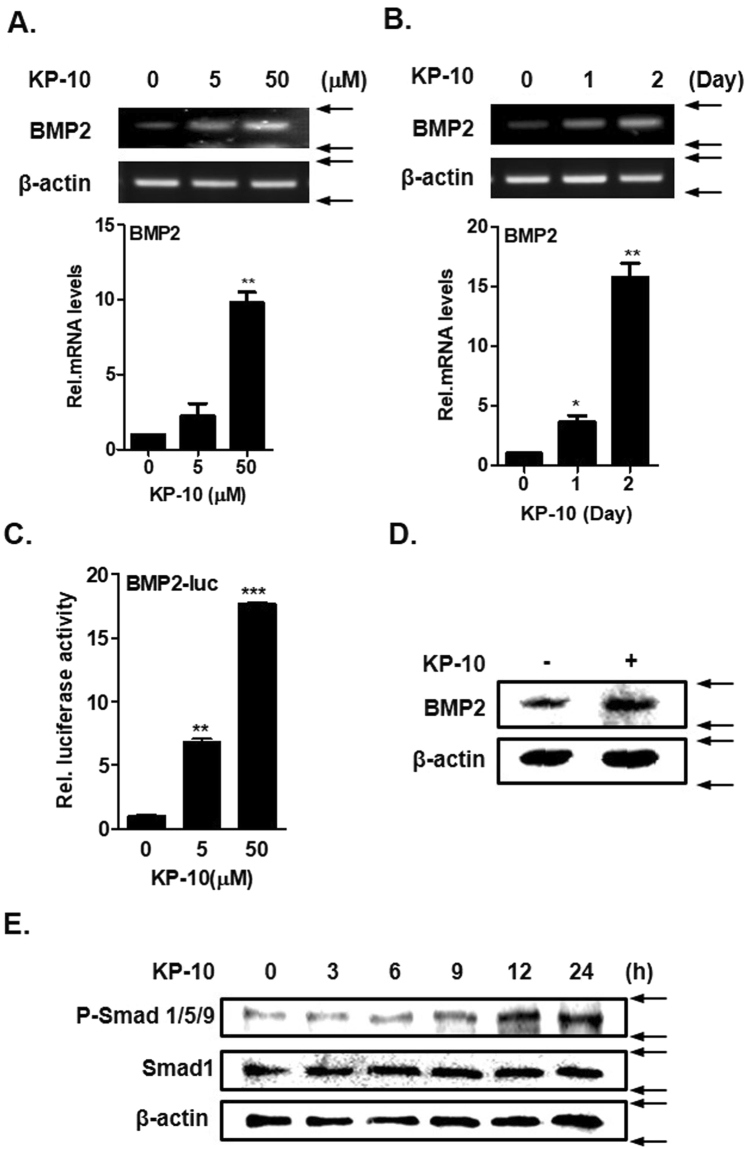
KP-10 increases BMP2 expression and Smad1/5/9 phosphorylation. (**A** and **B**) RT-PCR (upper panel) and real-time PCR (lower panel) were performed using total RNA isolated from cells treated with 5 or 50 μM KP-10 for 2 days (**A**) and 50 μM KP-10 for 1 and 2 days (**B**). *p < 0.05 and **p *<* 0.01 compared with the untreated control. Data represent the mean ± SEM of three individual experiments. (**C**) Cells were transfected with BMP2-luc (0.4 μg) for 6 h and then treated with KP-10 (5 or 50 μM). **p < 0.01 and ***p < 0.005 compared with the untreated control. Data represent the mean ± SEM of three individual experiments. (**D**) Cells were treated with 50 μM KP-10 for 4 days and harvested for Western blot analysis using the indicated antibodies. (**E**) C3H10T1/2 cells were treated with KP-10 for the indicated durations and harvested for Western blot analysis using the indicated antibodies.

BMP2 stimulates bone formation by activating Dlx5, Runx2, and ALP^27,28^. To determine whether KP-10 directly regulates BMP2 expression, C3H10T1/2 cells were treated with various doses of KP-10 for various durations. RT-PCR and real-time PCR analysis showed that KP-10 significantly increased the BMP2 mRNA level in a dose- and time-dependent manner (Fig. 2A and B). KP-10 also significantly increased BMP2-luc activity in a dose-dependent manner (Fig. 2C). Western blot analysis revealed that KP-10 increased the BMP2 protein level (Fig. 2D). Following extracellular secretion of BMP2, BMP2 dimers bind to BMP receptors in the cell membrane, which induces phosphorylation of Smad1/5/9^33^. KP-10 increased phosphorylation of Smad 1/5/9 in a time-dependent manner (Fig. 2E). This indicated that KP-10 increased BMP2 expression and activation. Taken together, these results suggest that KP-10 increased osteoblast differentiation by increasing expression and activation of BMP2 in C3H10T1/2 cells.

### KP-10 regulates BMP2 expression through NFATc4

**Figure 3:**
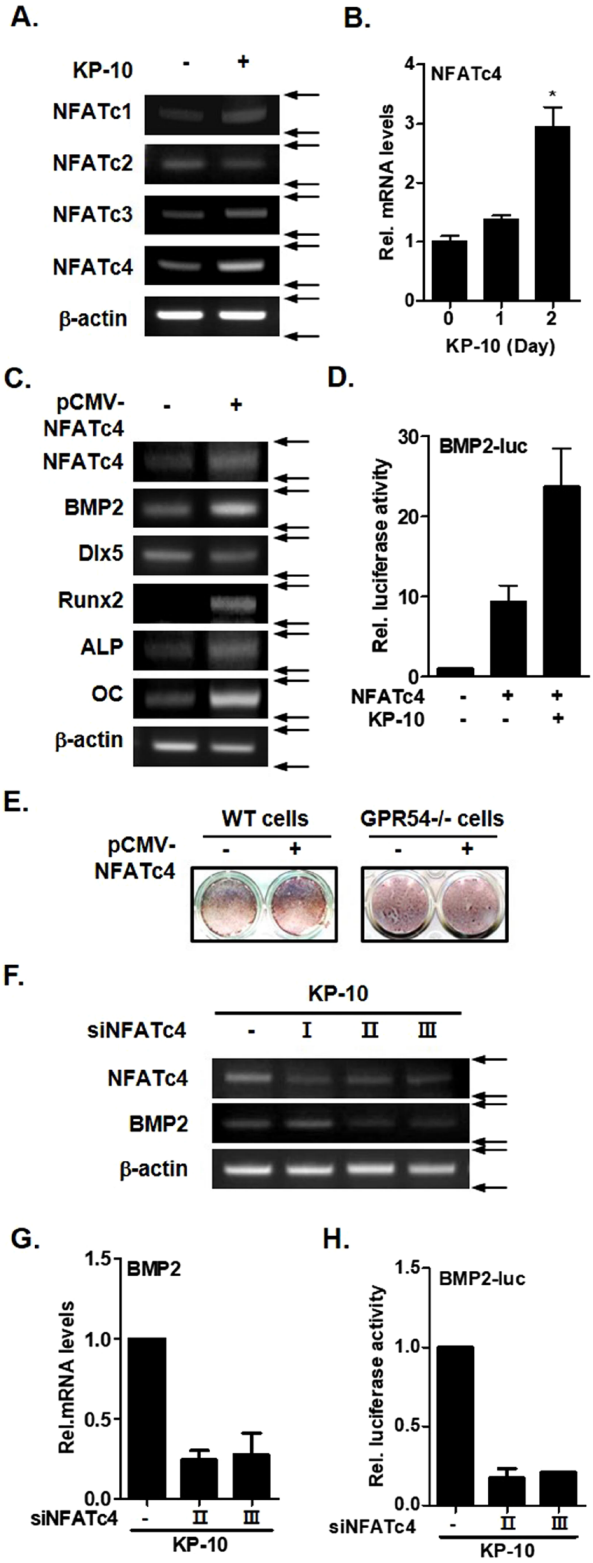
NFATc4 mediates KP-10-induced BMP2 expression. (**A**) RT-PCR was performed using total RNA isolated from cells treated with 50 μM KP-10 for 2days. (**B**) Real-time PCR was performed using total RNA isolated from cells treated with 50 μM KP-10 for 1 and 2 days. *p < 0.05 compared with the untreated control. Data represent the mean ± SEM of three individual experiments. (**C**) RT-PCR was performed using total RNA isolated from cells transfected with pCMV-NFATc4 (0.4 μg). (**D**) Cells were transfected with BMP2-luc (0.4 μg) and pCMV-NFATc4 (0.4 μg) for 6 h and then treated with KP-10 (+; 50 μM). (**E**) WT or GPR54^−/−^ C3H0T1/2 cells were cultured in the absence or pCMV-NFATc4 (0.4 μg). After 20 days, alizarin red S staining was performed to examine extracellular mineralization. (**F**) Cells were transfected with siNFATc4I, siNFATc4II or siNFATc4III for 6 h and then treated with KP-10 (+; 50 μM) for 2 days. Total RNA was isolated from the cell cultures, and RT-PCR analysis was performed with NFATc4, BMP2 and b-actin primers. (**G**) Real-time PCR was performed using total RNA isolated from cell transfected with siNFATc4II or siNFATc4III and then treated with 50 μM of KP-10 for 2 days. (**H**) Cells were co-transfected with BMP2-luc (0.4 μg) and siNFATc4 (2 or 3) for 6 h and then treated with KP-10 (+; 50 μM). I, II and III mean knockdown of NFATc4. These primers information is included in the materials and methods sessions.

KP-10 induces binding of dephosphorylated NFAT to the BMP7 promoter and thereby regulates BMP7 expression during kidney branching^13^. We investigated whether KP-10 induced expression of NFAT family members in osteoblasts. RT-PCR analyses demonstrated that KP-10 significantly increased expression of NFACTc4, whereas there were slight or no changes expression of NFATc1, NFATc3, and NFATc2 (Fig. 3A). KP-10 increased the NFATc4 mRNA level in a time-dependent manner (Fig. 3B). In addition, BMP2 and osteogenic genes expression was dramatically increased by NFATc4 overexpression using pCMV-NFATc4 (Fig. 3C). BMP2-luc activity was also increased by NFATc4 and/or KP-10 (Fig. 3D). To examine the effects of NFATc4 on mineralized nodule formation in osteoblasts alizarin red S staining performed. Overexpressed NFATc4 increased matrix mineralization in C3H10T1/2 cells. But, in GPR54^−/−^ cells was not affected (Fig. 3E). We constructed three types of NFATc4 siRNA primers (siNFATc4I, II and III). Among them, effect of siNFATc4II and III appeared, and next analysis was using the siNFATc4II and III. Interestingly, knockdown of NFATc4 did not increased BMP2 expression by KP-10 in C3H10T1/2 cells (Fig. 3F and G). In addition, siNFATc4 suppressed KP-10-induced BMP2-luc activity, as determined by a luciferase activity assay (Fig. 3H). Taken together, these results suggest that KP-10-induced the expression of the BMP2 gene via NFATc4.

### GPR54 deficiency suppresses KP-10-induced osteoblast differentiation

**Figure 4:**
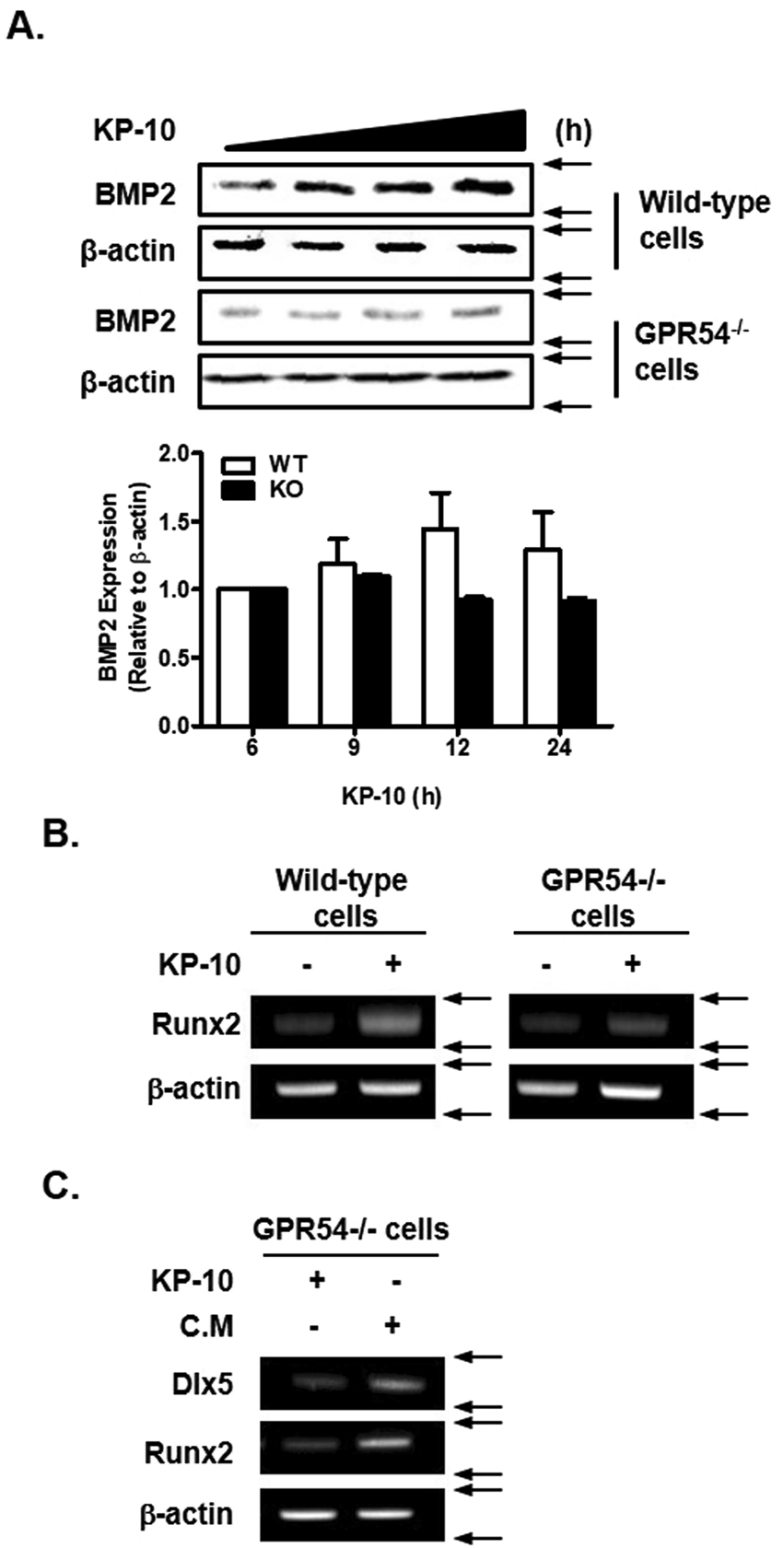
GPR54 is necessary for KP-10-induced osteoblast differentiation. (**A**) Wild-type and GPR54^−/−^ C3H10T1/2 cells were treated with 50 μM KP-10 for 6, 9, 12 or 24 h. Western blot analysis was performed using the indicated antibodies. BMP2 expression was normalized to β-actin expression. And the densitometry analysis was performed by ImageJ program (A, lower panel). (**B** and **C**) RT-PCR was performed using total RNA isolated from cultured cells. Wild-type and GPR54^−/−^ cells were treated with 50 μM KP-10 for 1 day (**B** and **C**). C.M of wild-type cells treated with 50 μM KP-10 for 12 h was collected. GPR54^−/−^ C3H10T1/2 cells were treated with 50 μM KP-10 or C.M for 2 days (**C**).

To investigate whether GPR54 is involved in KP-10-induced BMP2 expression, we analyzed BMP2 protein expression in wild-type and GPR54^−/−^ cells. KP-10 treatment increased BMP2 protein expression in wild-type cells, but not in GPR54^−/−^ cells (Fig. 4A). In addition, Runx2 (a downstream target gene of BMP2) expression was also not induced by KP-10 treatment in GPR54^−/−^ cells, whereas it was significantly increased in wild-type cells (Fig. 4B). Next, to examine whether KP-10 induces autocrine activity of BMP2 (BMP2 protein secreted to the media, and turn on the intracellular signaling by stimulation of specific receptor), we used the conditioned-medium (C.M) of C3H10T1/2 cells treated with KP-10 for 12 h. Expression of Dlx5 and Runx2 was increased in GPR54^−/−^ cells treated with C.M, but not in those treated with KP-10 (Fig. 4C). These results demonstrate that BMP2 protein has an autocrine effect upon KP-10 treatment. In summary, GPR54 mediated KP-10-induced BMP2 expression, and BMP2 increased expression of osteogenic genes such as Dlx5 and Runx2 via a KP-10-induced autocrine effect in C3H10T1/2 cells.

## Discussion

In this study, we first demonstrated that KP-10 stimulated osteoblast differentiation through the BMP2 signaling pathway in C3H10T1/2 cells. Our data provide evidence that KP-10/GPR54 signaling induces osteoblast differentiation via NFATc4-mediated BMP2 expression and activation.

KP-10 is important for the regulation of tumor metastasis, puberty onset, and fertility^3,28,29^. KP-10 and its receptor, GPR54, are key components in the regulation of GnRH secretion in humans and other mammals^11,27^. However, the role of KP-10/GPR54-mediated signaling in osteoblast differentiation is unknown. Therefore, we focused on the role of KP-10 in osteoblasts. Osteoblasts express various phenotypic markers, including Dlx5, Runx2, and ALP^23–25^. BMPs play an instrumental role in osteoblast differentiation signaling, and their effects are mediated through Smad signaling^30,31^. KP-10 positively regulated osteoblast differentiation in C3H10T1/2 cells. In addition, KP-10 significantly increased expression of mature osteoblast marker genes (Dlx5, Runx2, and ALP) and ALP activity. Moreover, KP-10 increased BMP2 gene and protein expression. These results suggest that KP-10 regulates osteoblast differentiation though BMP2 in C3H10T1/2 cells.

To gain insights into the mechanisms by which KP-10 regulates osteoblast differentiation, we first evaluated BMP2 signaling because the BMP2-pSmad-Dlx5/Runx2 pathway is involved in osteoblast differentiation^34–37^. BMP2 regulates Runx2 by activating Smad 1/5/9^26,27^. BMP2 further regulates transcription of osteogenic genes, including Dlx5^28^. KP-10 significantly increased phosphorylation of Smad 1/5/9 in a time-dependent manner as well as expression of the osteogenic markers Runx2 and Dlx5. These results suggest that KP-10-induced osteoblast differentiation is mediated by Smad 1/5/9 and BMP2 signaling.

The role of KP-10/GPR54-mediated signaling in the BMP2 signaling pathway is unknown. GPR54 regulates BMP7 expression through NFATc2 and Sp1, and plays an important role in embryonic kidney branching morphogenesis and glomerular development^13^. Interaction of KP-10, estrogen and BMP4 regulates GnRH production in GT1-7 cells^14^. In this study, KP-10 regulated BMP2 expression through NFATc4 while osteoblast differentiation. The transcription factor NFATc4 is involved in BMP2 transcriptional regulation. Activation of GPR54 by KP-10 led to binding of NFATc4 to the BMP2 promoter. In addition, the KP-10/GPR54 interaction was important for osteoblast differentiation.

**Figure 5:**
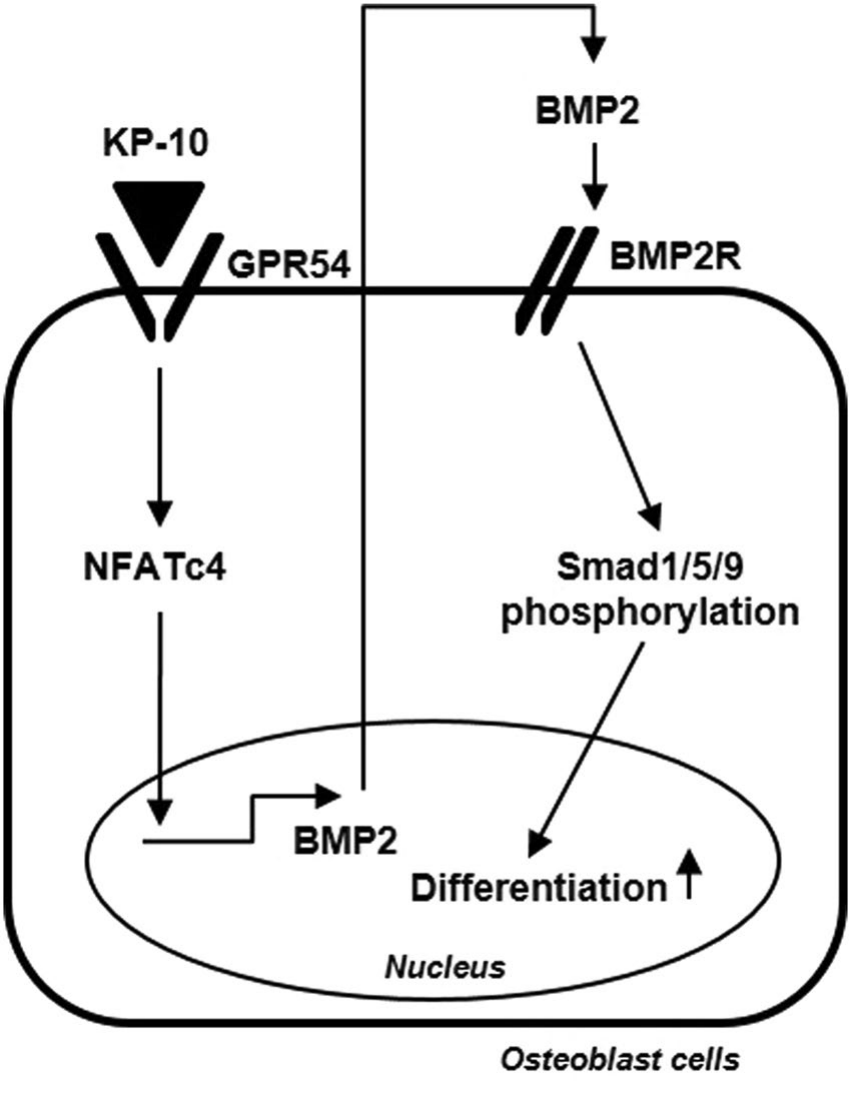
Overview of KP-10-induced osteoblast differentiation. KP-10/GPR54 signaling induces BMP2 expression via NFATc4. And the autocrine effect of BMP2 increased the expression of osteogenic genes expressions through Smad1/5/9 phosphorylation.

In summary, this study demonstrated that KP-10 induces osteoblast differentiation in C3H10T1/2 cells. We propose that KP-10/GPR54 signaling induced osteoblast differentiation via NFATc4-mediated BMP2 expression. The autocrine effect of BMP2 increased the expression of osteogenic genes through Smad1/5/9 phosphorylation (Fig. 5).

## Materials and Methods

### Reagents and antibodies

KP-10 (AS-64684) was purchased from ANASPEC Co. (Fremont, CA). Dulbecco’s modified eagle medium (DMEM), phosphate buffered saline, penicillin streptomycin, and 0.25% trypsin-EDTA were purchased from GIBCO-BRL (Grand Island, NY). Fetal bovine serum (FBS) was purchased from MP Biomedicals (Seoul, Korea). Emerald Amp^®^ GRPCR Master Mix was purchased from TaKaRa (Shiga, Japan), and AmpiGene^TM^ qPCR Green Mix Hi-ROX was purchased from Enzo (Farmingdale, NY). Recombinant human BMP2 was purchased from Cowellmedi Co. (Busan, Korea). Antibodies against BMP2 and β-actin were from Santa Cruz Biotechnology (Dallas, TX). Antibodies against Smad and phospho-Smad (p-Smad) were from Cell Signaling Technology (Cambridge, MA).

### Cell culture

The mouse mesenchymal stem cell line C3H10T1/2 (ATCC, Manassas, VA) was maintained in DMEM containing 10% FBS, 100 units/mL penicillin and 100 μg/mL streptomycin in humidified air containing 5% CO_2_ at 37 °C. Differentiation of osteoblasts was induced by addition of osteogenic medium containing 2% FBS, 50 μg/mL ascorbic acid (Sigma Aldrich, St. Louis, MO) and 5 mM β-glycerophosphate (Sigma Aldrich). The culture medium was replaced every 2 days. To evaluate the effect of KP-10, cells were cultured in medium containing 50 mM KP-10.

### RT-PCR and real-time PCR analysis

Total RNA was isolated from cells using TRIzol reagent (Bio Science Technology, Daegu, Korea) as per the manufacturer’s instructions. Reverse transcription was performed using 1 μg of total RNA. RT-PCR conditions were initial denaturation at 95 °C for 5 min followed by a three-step cycle of denaturation at 95 °C for 30 s, annealing at the optimal temperature for each primer pair for 30 s, and extension at 72 °C for 30 s. After 30–35 cycles, a final extension was performed at 72 °C for 5 min. The RT-PCR primer sequences were as follows: β-actin forward, 5′-TTCTTTGCAGCTCCTTCGTTGCCG-3′; β-actin reverse, 5′-TGGATGGCTACGTACATGGCTGGG-3′; Dlx5 forward, 5′-CAGAAGAGTCCCAAGCATCC-3′; Dlx5 reverse, 5′-GAGCGCTTTGCCATAAGAAG-3′; Runx2 forward, 5′-CCGCACGACAACCGCACCAT-3′; Runx2 reverse, 5′-CGCTCCGGCCCACAAATCTC-3′; ALP forward, 5′-ATCTTTGGTCTCGCTCCCATG-3′; ALP reverse, 5′-TTTCCCGTTCACCGTCCAC-3′; OC forward, 5′-GGCAGCGAGGTAGTGAAG-3′; OC reverse, 5′-CGTAGAAGCGCCGATAGG-3′; BMP2 forward, 5′-ACCAGACTATTGGACACCAG-3′; BMP2 reverse, 5′-AATCTCACATGTCTCTTGG-3′; NFATc1 forward, 5′-CCTTCGGAAGGGTGCCTTTT-3′; NFATc1 reverse, 5′-AGGCGTGGGGCCTCAGCAGG-3′; NFATc2 forward, 5′-TGGCCCGCGACATCTACCCT-3′; NFATc2 reverse, 5′-TGGTAGAAGGCGTGCGGCTT-3′; NFATc3 forward, 5′-TGGATCTCAGTATCCTTTAA-3′; NFATc3 reverse, 5′-CACACGAAATACAAGTCGGA-3′; NFATc4 forward, 5′-CATTGGCACTGCAGATGAG-3′; and NFATc4 reverse, 5′-CGTAGCTCAATGTCTGAAT-3′. Real-time PCR was performed using 1 μg of total RNA. Each reaction consisted of initial denaturation at 95 °C for 5 min, followed by a three-step cycle of denaturation at 95 °C for 30 s, annealing at the optimal temperature for each primer pair for 30 s, and extension at 72 °C for 30 s. After 45 cycles, a final extension was performed at 72 °C for 5 min. The real-time PCR primer sequences were as follows: β-actin forward, 5′-TTCTTTGCAGCTCCTTCGTTGCCG-3′ β-actin reverse, 5′-TGGATGGCTACGTACATGGCTGGG-3′; Id-1 forward, 5′-CTTCAGGAGGCAAGAGGAAA-3′; Id-1 reverse, 5′-CAAACCCTCTACCCACTGGA-3′; Dlx5 forward, 5′-GCCCACCAACCAGCCAGAGA-3′; Dlx5 reverse, 5′-GCGAGGTACTGAGTCTTCTGAAACC-3′; Runx2 forward, 5′-AGATGACATCCCCATCCATC3′; Runx2 reverse, 5′-GTGAGGGATGAAATGCTGG-3′; ALP forward, 5′-AACCCAGACACAAGCATTCC-3′; ALP reverse, 5′-GAGAGCGAAGGGTCAGTCAG-3′; OC forward, 5′-CTGACCTCACAGATGCCAAG-3′; OC reverse, 5′-GTAGCGCCGGAGTCTGTTC-3′; BMP2 forward, 5′-AAGCGTCAAGCCAAACACAAAC-3′; BMP2 reverse, 5′-GCCACGATCCAGTCATTCCAC-3′; NFATc4 forward, 5′-ATCACTGGCAAGATGGTGGCTACA-3′; and NFATc4 reverse, 5′-AGCTTCAGGATTCCAGCACAGTCA-3′. Expression levels were normalized to those of endogenous β-actin and data were analyzed using the ^ΔΔ−^C_T_ method^26^.

### Transient transfection and the luciferase assay

C3H10T1/2 cells were transiently transfected with the indicated plasmids using Lipofectamine 2000 (Invitrogen, Carlsbad, CA) as described previously^22^. Cells were harvested 48 h after transfection. Luciferase activity was measured using a luciferase reporter assay system (Promega, Madison, WI) and a luminometer as per the manufacturer’s instructions.

### Silencing of NFATc4

The siRNA for NFATc4 were synthesized chemically (Bioneer, Daejeon, Korea), deprotected, annealed, and transfected according to the manufacturer’s instructions. The C3H10T1/2 cells were transfected with the siRNAs using Lipofectamine 2000 (Invitrogen, Carlsbad, CA). The sequences of siRNA ae as follows: siNFATc4I sense 5′-GUGUUGACUGGUUCCAACU-3′; siNFATc4I antisense 5′-AGUUGGAACCAGUCAACAC-3′; siNFATc4II sense 5′-CUACUUUUACGUCUCCAAU-3′; siNFATc4II antisense 5′-AUUGGAGACGUAAAAGUAG-3′; siNFATc4III sense 5′-CAGAACUGACUGGGCUGAA-3′; siNFATc4III antisense 5′-UUCAGCCCAGUCAGUUCUG-3′.

### ALP staining

C3H10T1/2 was cultured with ascorbic acid (50 μg/mL), β-glycerophosphate (5 mM) and KP-10 (50 μM) for 4 days. Staining was performed using standard protocols. Briefly, cultured cells were fixed with 10% formaldehyde, rinsed twice with deionized water, and treated with BCIP^®^/NBT solution (Sigma Aldrich) for 15 min. After additional washing, stained cultures were imaged.

### Alizarin red S staining

Alizarin red S (Sigma-Aldrich) was solubilized with distilled water. For mineralization analysis, C3H10T1/2 cells were treated with 50 μM KP-10 or transfected with pCMV-NFATc4 (0.4 g) for 20 days and then fixed with 4% formaldehyde (Duksan Pure chemicals Inc.) for 5 min. After washing with distilled water, cells were stained with 300 μg/mL of Alizarin red S solution for 30 min at room temperature. And washing with distilled water. Staining was then documented with an Epson perfection V37 scanner (Seiko Epson, Suwa, Japan).

### Western blot analysis

Total cells were harvested using an EzRIPA Lysis kit (ATTO Technology, Tokyo, Japan) and then centrifuged at 12,000 g for 10 min at 4 °C. Total proteins were quantified using the Bradford assay, separated by SDS-PAGE, and transferred to a PVDF membrane. After blocking in 5% skimmed milk prepared in Tris-buffered saline containing Tween 20, the membrane was incubated with specific primary antibodies (1:1000). Signals were detected using ECL reagent (Advansta, Menlo Park, CA). Densitometric analysis of the blotted membrane was performed using a FUSION solo analyzer system (Vilber Lourmat, Eberhardzell, Germany).

### CRISPR/Cas9 plasmid for GPR54

The Cas9-expressing plasmid was purchased from Addgene (Cambridge, MA). The sgRNA plasmid, which expresses crRNA and tracrRNA under the control of the hU6 promoter was subcloned from the pCLIIP-ALL-EFS-Puro cloning vector (TransOMIC technologies, Huntsville, AL) into the minimal PUC18 backbone plasmid. To knock out the GPR54 gene, oligonucleotides containing target sequences for exon1 were synthesized (Bioneer, Daejeon, Korea) and inserted into the sgRNA plasmid that had been digested with BsmBI.

### Transfection and the T7E1 assay

Cells were transfected using the 4D-nucleofector system (Amaxa, Koeln, Germany) at a molecular weight ratio of 1:2 (plasmid encoding Cas9: plasmid encoding sgRNA) and 700 cells were spread over a 100 mm culture dish to form single cell-derived colonies. Then, 5–10 cells from a colony were collected and lysed. Mutant colonies were selected via PCR and the T7E1 assay. Nested-PCR to amplify the GPR54 gene including the exon1 region was performed with DNA-specific primers (forward, 5′-CAGGACACAATCCTTGAAGG-3′; reverse (1), 5′-GTAGGAAAGTGACGTCTGTG-3′; and reverse (2), 5′-CTCGCTTCGTTCCTGACTTG-3′). PCR products were denatured at 95 °C and re-annealed by reducing the temperature to randomly generate heteroduplex DNA, which was treated with 5 units of T7 endonuclease 1 (New England Biolabs, Beverly, MA) for 1 h at 37 °C and analyzed using 2% agarose gel electrophoresis.

### Statistical analysis

All experiments were repeated at least three times. Statistical analysis was performed using the Student’s *t*-test or analysis of variance, followed by Duncan’s multiple comparison tests. P values of < 0.05 were considered significant. Results are expressed as the mean ± SEM of triplicate independent samples.

## References

[1] Kotani M (2001). The metastasis suppressor gene KiSS-1 encodes kisspeptins, the natural ligands of the orphan G protein-coupled receptor GPR54. The Journal of biological chemistry.

[2] Muir AI (2001). AXOR12, a novel human G protein-coupled receptor, activated by the peptide KiSS-1. The Journal of biological chemistry.

[3] Ohtaki T (2001). Metastasis suppressor gene KiSS-1 encodes peptide ligand of a G-protein-coupled receptor. Nature.

[4] Lee JH (1996). KiSS-1, a novel human malignant melanoma metastasis-suppressor gene. Journal of the National Cancer Institute.

[5] Cvetkovic D, Babwah AV, Bhattacharya M (2013). Kisspeptin/KISS1R System in Breast Cancer. Journal of Cancer.

[6] Lee JH, Welch DR (1997). Suppression of metastasis in human breast carcinoma MDA-MB-435 cells after transfection with the metastasis suppressor gene, KiSS-1. Cancer research.

[7] Olbrich T (2010). Kisspeptin-10 inhibits bone-directed migration of GPR54-positive breast cancer cells: Evidence for a dose-window effect. Gynecologic oncology.

[8] Stafford LJ, Xia C, Ma W, Cai Y, Liu M (2002). Identification and characterization of mouse metastasis-suppressor KiSS1 and its G-protein-coupled receptor. Cancer research.

[9] Beier DR, Dluhy RG (2003). Bench and bedside–the G protein-coupled receptor GPR54 and puberty. The New England journal of medicine.

[10] d’Anglemont de Tassigny X (2007). Hypogonadotropic hypogonadism in mice lacking a functional Kiss1 gene. Proceedings of the National Academy of Sciences of the United States of America.

[11] de Roux N (2003). Hypogonadotropic hypogonadism due to loss of function of the KiSS1-derived peptide receptor GPR54. Proceedings of the National Academy of Sciences of the United States of America.

[12] Teles MG (2008). A GPR54-activating mutation in a patient with central precocious puberty. The New England journal of medicine.

[13] Yi T (2010). Regulation of embryonic kidney branching morphogenesis and glomerular development by KISS1 receptor (Gpr54) through NFAT2- and Sp1-mediated Bmp7 expression. The Journal of biological chemistry.

[14] Terasaka T (2013). Mutual interaction of kisspeptin, estrogen and bone morphogenetic protein-4 activity in GnRH regulation by GT1-7 cells. Molecular and cellular endocrinology.

[15] Crabtree GR, Olson EN (2002). NFAT signaling: choreographing the social lives of cells. Cell.

[16] Rao A, Luo C, Hogan PG (1997). Transcription factors of the NFAT family: regulation and function. Annual review of immunology.

[17] Hogan PG, Chen L, Nardone J, Rao A (2003). Transcriptional regulation by calcium, calcineurin, and NFAT. Genes & development.

[18] Wu H, Peisley A, Graef IA, Crabtree GR (2007). NFAT signaling and the invention of vertebrates. Trends in cell biology.

[19] Winslow MM (2006). Calcineurin/NFAT signaling in osteoblasts regulates bone mass. Developmental cell.

[20] Chen, S. & Pan, M. *NFAT signaling and bone homeostasis*. *Journal of Hematology & Thromboembolic Diseases* (2013).

[21] Takayanagi H (2002). Induction and activation of the transcription factor NFATc1 (NFAT2) integrate RANKL signaling in terminal differentiation of osteoclasts. Developmental cell.

[22] Koga T (2005). NFAT and Osterix cooperatively regulate bone formation. Nature medicine.

[23] Yamaguchi A, Komori T, Suda T (2000). Regulation of osteoblast differentiation mediated by bone morphogenetic proteins, hedgehogs, and Cbfa1. Endocrine reviews.

[24] Komori T (2006). Regulation of osteoblast differentiation by transcription factors. Journal of cellular biochemistry.

[25] Szulc P, Garnero P, Marchand F, Duboeuf F, Delmas PD (2005). Biochemical markers of bone formation reflect endosteal bone loss in elderly men–MINOS study. Bone.

[26] Ryoo HM, Lee MH, Kim YJ (2006). Critical molecular switches involved in BMP-2-induced osteogenic differentiation of mesenchymal cells. Gene.

[27] Javed A (2008). Structural coupling of Smad and Runx2 for execution of the BMP2 osteogenic signal. The Journal of biological chemistry.

[28] Jang WG, Kim EJ, Lee KN, Son HJ, Koh JT (2011). AMP-activated protein kinase (AMPK) positively regulates osteoblast differentiation via induction of Dlx5-dependent Runx2 expression in MC3T3E1 cells. Biochemical and biophysical research communications.

[29] Ducy P (1999). A Cbfa1-dependent genetic pathway controls bone formation beyond embryonic development. Genes & development.

[30] Javed A (2001). runt homology domain transcription factors (Runx, Cbfa, and AML) mediate repression of the bone sialoprotein promoter: evidence for promoter context-dependent activity of Cbfa proteins. Molecular and cellular biology.

[31] Lee MH (2003). BMP-2-induced Runx2 expression is mediated by Dlx5, and TGF-beta 1 opposes the BMP-2-induced osteoblast differentiation by suppression of Dlx5 expression. The Journal of biological chemistry.

[32] Whyte MP, Vrabel LA (1987). Infantile hypophosphatasia fibroblasts proliferate normally in culture: evidence against a role for alkaline phosphatase (tissue nonspecific isoenzyme) in the regulation of cell growth and differentiation. Calcified tissue international.

[33] Sedlmeier G, Sleeman JP (2017). Extracellular regulation of BMP signaling: welcome to the matrix. Biochemical Society transactions.

[34] Brown RE, Imran SA, Ur E, Wilkinson M (2008). KiSS-1 mRNA in adipose tissue is regulated by sex hormones and food intake. Molecular and cellular endocrinology.

[35] Ghosh-Choudhury N (2002). Requirement of BMP-2-induced phosphatidylinositol 3-kinase and Akt serine/threonine kinase in osteoblast differentiation and Smad-dependent BMP-2 gene transcription. The Journal of biological chemistry.

[36] Hauge-Evans AC (2006). A role for kisspeptin in islet function. Diabetologia.

[37] Phimphilai M, Zhao Z, Boules H, Roca H, Franceschi RT (2006). BMP signaling is required for RUNX2-dependent induction of the osteoblast phenotype. Journal of bone and mineral research: the official journal of the American Society for Bone and Mineral Research.

